# The Effect of the Activation Process and Metal Oxide Addition (CaO, MgO, SrO) on the Catalytic and Physicochemical Properties of Natural Zeolite in Transesterification Reaction

**DOI:** 10.3390/ma14092415

**Published:** 2021-05-06

**Authors:** Pawel Mierczynski, Lukasz Szkudlarek, Karolina Chalupka, Waldemar Maniukiewicz, Satriyo K. Wahono, Krasimir Vasilev, Malgorzata I. Szynkowska-Jozwik

**Affiliations:** 1 Institute of General and Ecological Chemistry, Faculty of Chemistry, Lodz University of Technology, Zeromskiego 116, 90-924 Lodz, Poland; lukasz.szkudlarek@dokt.p.lodz.pl (L.S.); karolina.chalupka@p.lodz.pl (K.C.); waldemar.maniukiewicz@p.lodz.pl (W.M.); malgorzata.szynkowska@p.lodz.pl (M.I.S.-J.); 2 Research Division for Natural Product Technology, Indonesian Institutes of Sciences, Jl. Jogja–Wonosari km 32, Gading, Playen, Gunungkidul, Yogyakarta 55861, Indonesia; satriyo.krido.wahono@lipi.go.id; 3 Academic Unit of STEM, University of South Australia, Mawson Lakes, Adelaide, SA 5095, Australia; krasimir.vasilev@unisa.edu.au

**Keywords:** biodiesel production, metal-oxide catalyst, zeolite, FAME

## Abstract

This work provides valuable information about unexplored catalytic systems tested in the transesterification reaction of vegetable oil with methanol. It was demonstrated that natural zeolite treatment leads to enhanced catalytic activity and yield of biodiesel production. The activation of the catalytic material in a mixture of 5% H_2_–95% Ar resulted in an improvement of the values of the TG conversion and fatty acid methyl esters (FAME) yield. In addition, it was proven that the incorporation of CaO, MgO and SrO oxides onto the natural zeolite surface improves the TG conversion and FAME yield values in the transesterification reaction.

## 1. Introduction

Increased energy production is a key requirement for a given country’s economic development. The continuing growth of the world population and economic development results in a growing demand for fuel [1,2]. Today, fossil fuels are still the main source of energy used to meet energy needs [3]. The constantly growing consumption of fossil fuels and depletion of resources is driving the prices up. In addition, the increase in greenhouse gas emissions caused by fossil fuel combustion causes significant environmental pollution [4]. All of this demonstrates the urgent need to search for new, sustainable and environmentally friendly alternatives to fossil fuels [3,5,6,7,8,9,10]. One of the main alternative fuels is biodiesel. Biodiesel is a carbon-neutral fuel source that can provide almost unlimited renewable energy [1,11,12,13]. It has been recognized as an alternative fuel with similar properties to traditional diesel fuel [4]. Moreover, biodiesel has several advantages. Biodiesel is a biodegradable fuel produced from renewable feedstocks such as waste cooking oil, vegetable oils, or animal fats, making it possible to use the excess of these raw materials for use in its production [2,14,15].

Utilization of biodiesel means a cleaner combustion process for this fuel combined with lower emissions of unburned hydrocarbons, binders, polyaromatics, smoke and noise. At the same time, when burning biodiesel, there is no introduction of additional amounts of carbon dioxide into the environment. In addition, biodiesel has a higher cetane number than traditional diesel fuel, high flash point and provides better lubrication. Biodiesel can be used as an additive to conventional diesel in the form of a blend labeled “BX”, where “X” is the percentage of biodiesel in the blend, for example, “B5” combines 5% biodiesel and 95% petrodiesel, “B20” is 20% biodiesel and 80% traditional diesel fuel (such a blend is called biosolar). At the same time, “B100” stands for pure biodiesel. Chemically, biodiesel is a mixture of monoalkyl esters of long-chain fatty acids [16,17,18,19], which can be produced by emulsification, pyrolysis, transesterification, cracking [8], or direct esterification of the free fatty acid. Among these production methods, the transesterification of vegetable oils or animal fats is the most preferred biodiesel production method due to the high yields obtained during the reaction carried out under mild conditions. The other methods have several disadvantages, such as heavy carbon deposits, incomplete combustion, increase of lubricating oil viscosity and undesirable byproducts, such as unsaturated hydrocarbons (alkenes, alkadienes), alkanes, aromatic and carboxylic acids.

**Scheme 1:**
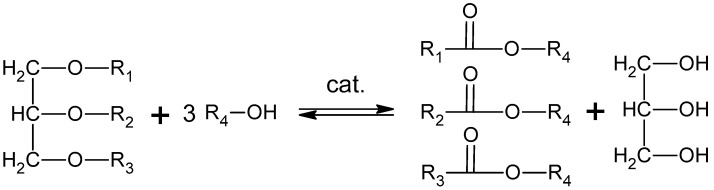
Scheme of the transesterification reaction.

The transesterification of vegetable oils is the reaction of triglycerides from the oil with short-chain alcohols (see Scheme 1).

The reaction produces glycerol and a mixture of fatty acid esters. The stoichiometry of the reaction shows that 3 moles of alcohol and 1 mole of triglyceride are required to carry out the transesterification process, which gives 3 moles of fatty acids methyl esters (FAME) and 1 mole of glycerol [20,21]. Methanol is often used for the reaction due to its physicochemical nature to produce fatty acid methyl esters (FAME) [20,22,23]. The use of ethanol in the reaction is increasing in popularity due to the renewable nature of lower toxicity than methanol [1]. Biodiesel obtained as a result of transesterification (methyl or ethyl esters of fatty acids) consists of less massive molecules [20], which with the appropriate viscosity and combustion rate (cetane number) can be used in existing diesel engines with compressors [1]. Apart from the production of biodiesel, the transesterification of vegetable oils with methanol can be used for the production of raw materials for the chemical industry, for example, for the production of isopropyl esters and fatty alcohols, or for the production of non-ionic biodegradable surfactants such as alkanolamides and alkyl glucosides [24]. In practice, transesterification is carried out in the presence of a catalyst, either homogeneous or heterogeneous [25]. To carry out the reaction in mild conditions and to control the product selectivity, this process is carried out with a homogeneous catalyst, either acid catalysts, e.g., sulfuric acid (H_2_SO_4_) [1], hydrochloric acid (HCl) [26] or base catalysts, e.g., sodium hydroxide (NaOH), potassium hydroxide (KOH) [24], sodium methoxide (CH_3_ONa), potassium methoxide (CH_3_OK) [3]. Alkali-catalyzed transesterification is much faster than the acid-catalyzed reaction. Therefore, base catalysts are most commonly used commercially [20]. Homogeneous catalysis as a method of biodiesel production still dominates despite high costs, many limitations and disadvantages. In this method, all reactants, products and catalysts are in one phase, making the separation of the catalyst from the reaction product difficult, if not impossible, to recover and regenerate. In addition, a large amount of toxic wastewater is generated in the biodiesel purification process. Therefore, attention is paid to the replacement of homogeneous catalysis by using heterogeneous catalysts [24]. Switching to heterogeneous catalysts in biodiesel production has many advantages. It is an economical and environmentally friendly solution producing less toxic waste [3] and releasing less pollutants into the environment, which overall reduces the total production costs. Heterogeneous catalysts are more active and show high potency when using lower temperatures and/or shorter reaction times. As the reactants and products are in a phase different than the catalyst, separating the catalyst from the products is easy and enables regeneration and reuse. Heterogeneous catalysts are less corrosive than their homogeneous counterparts, are easier to handle and are strong, long-lasting and durable [25]. Examples of heterogeneous catalysts used in the transesterification of vegetable oils are alkaline earth metal oxides and derivates (CaO, MgO, SrO), boron and carbon group elements (generally loaded on alumina) [27], amorphous zirconium oxide, zeolites, titanium and potassium zirconia’s [11], sulphated oxides (SO_4_^2−^/ZrO_2_), cation-exchanged resins, tungstated zirconia–alumina (WZA—e.g., WO_3_/ZrO_2_) [27]. Besides of the type of catalyst, there are several aspects that affect the reaction rate of transesterification, i.e., temperature, purity of the reactants (mainly water content), free fatty acid content, alcohol to oil molar ratio, process time [28].

Zeolites are crystalline aluminosilicates forming three-dimensional skeletal structures, mostly channels and pores, providing zeolites with a large surface area. The structure consists of three-dimensional frameworks of SiO_4_ and AlO_4_ tetrahedral. Generally, the structure of zeolites is formed from the primary unit, in the form of tetrahedral, which then becomes a secondary unit of the polyhedral of a zeolite structure unit [29,30,31]. Each pore and the inner zeolite channel and between the crystals are considered cylindrical, so the total pore area is the accumulation of pore surface (wall) and zeolite constituent channels and depends on their number—the greater the number of pores, the greater the total surface area of the material. The inner surface of the zeolite may be up to two orders of magnitude larger than the outer one. Activation of zeolite can be done physically by reducing the grain size, sieving and heating at high temperatures, which removes organic impurities, enlarges the pores, and increases the surface; and chemically, adding acid causes the exchange of H^+^ ions [29]. The general empirical formula describing the chemical structure of this material is as follows: M_2n_O ∙ Al_2_O_3_ ∙ x SiO_2_ ∙ y H_2_O, where *M* stands for any alkaline or alkaline earth cation, *n* is the valence of the cation, *x* varies from 2 to 10, and *y* varies from 2 to 7. Several types of zeolite backbones are recognized, i.e., porous, canal, zigzag, etc. [20]. Due to their chemical properties, zeolites are widely used in many fields, including industrial (e.g., water treatment and purification, humidity control), as molecular sieves, selective absorbent, or ion exchange resins or in heterogeneous catalysis as a component of high-activity catalysts or as a catalyst itself [3].

Zeolites are characterized by their acidic sites and shape selectivity in catalysis. The acidity of zeolites comes from the charge imbalance between silica and alumina units bound in a stable aluminosilicate crystal structure [32]. Zeolites can be obtained by synthesis, e.g., by heating a solution of some form of silica, alumina, and alkylammonium salts in water, which over time forms a solid precipitated aluminosilicate zeolite. Moreover, zeolite can be obtained from components of natural origin, i.e., clay, rocks and ash residues from solid fuel combustion, containing large amounts of oxygen, silicon and aluminum with a similar chemical composition to zeolites [4]. Natural zeolites (NZ) are an abundant and low-cost resource [25]. Due to the porous structure with the capacity to accommodate small molecules (such as triglycerides) and chemical and thermal stability, zeolites are considered potential catalysts for biodiesel production [25]. By making modifications to natural zeolites, the catalytic activity of the material can be increase, leading to higher biodiesel efficiency. Zeolites can be modified by impregnation with an acid or a base [25]. However, previous research shows that alkali impregnation is a better method for zeolite modification than acid impregnation [33]. Zeolite catalysts must be resistant to a high content of free fatty acids and, at the same time, be water-resistant and show high efficiency and effectiveness in the transesterification reaction. The disadvantage of the natural zeolites is the presence of impurities in the form of various intercalates and exchangeable metals and their oxides, the presence of which may increase or decrease the catalytic capacity of the material. Hence, for a long time, transesterification as a biodiesel production method was catalyzed using synthetic zeolites, which are pure materials [20]. Typical natural zeolite-containing systems extensively used in biodiesel reaction include; K_2_CO_3_/natural zeolite from North Sumatra, Indonesia, with rice bran oil [25]; KOH impregnated natural zeolite from Banten Province, natural zeolite from Tehran, Iran activated by KOH with waste cooking oil; CaO/natural zeolite from Banten Province, Indonesia with waste cooking oil [34]; KOH/natural zeolite from Bandung with castor oil [35]; H-natural zeolite from Ujung Pancu, Aceh Besar/KI with crude palm oil [4]; and La/natural zeolite activated by HCl with crude palm oil, natural zeolite from Gilgil, Kenya modified by Na, Cu, Pb in an amount of 0.1 mol of the cation over 50 g material with jatropha oil [20].

The main goal of this work was to determine the influence of the catalytic material (NZ) activation process on its catalytic and physicochemical properties in the transesterification reaction of rapeseed oil with methanol. Furthermore, the addition effect of CaO, MgO or SrO oxides on the catalytic reactivity of natural zeolite in biodiesel production was also studied. The physicochemical properties of the treated natural zeolite and the metal-oxide catalysts were extensively investigated by CO_2_-TPD, XRD, FTIR and SEM-EDS. The obtained results were used to explain the differences in the catalytic activity of the materials in the transesterification reaction of vegetable oils with methanol.

## 2. Materials and Methods

### 2.1. Preparation of the Catalytic Materials

The 10% CaO, 10% MgO, and 10% SrO metal-oxide catalysts were synthesized by impregnation method using natural zeolite samples as support (for each sample, we introduced the same molar quantity of the metal oxide). Natural zeolite (calcite-rich mordenite–clinoptilolite zeolite) from Gunungkidul (Yogyakarta, Indonesia) used in this study was supplied as a powder by a local mining supplier company, CV Mountain Stone) [36,37]. In addition, natural zeolite was activated under various conditions to study the physicochemical properties and catalytic activity in the transesterification reaction. The NZ sample was calcined in an air atmosphere for 4 h at 400 °C or reduced in a mixture of 5% H_2_–95% Ar mixture at 300, 400 or 500 °C for 1 h. During the preparation of the metal-oxide catalysts, the precursors of the metallic oxide phase such as calcium, magnesium and strontium nitrates (Sigma-Aldrich, Poznan, Poland) were used to synthesize the catalytic systems. The metal oxide-loading in all cases was 10 wt %. The synthesized metal-oxide catalysts were dried for 2 h at 120 °C and then calcined in an air atmosphere at 400 °C for 4 h.

### 2.2. Characterization of the Catalytic Material

The catalyst surface morphology was investigated using an S-4700 scanning electron microscope HITACHI (Tokyo, Japan), equipped with an energy dispersive spectrometer (ThermoNoran, Madison, WI, USA) (SEM-EDS). The phase composition studies of supports and catalysts were investigated using the X-ray diffraction technique (XRD). To study, the X-ray diffraction patterns were recorded on a PANalytical X’PertPRO MPD diffractometer in Bragg–Brentano reflecting geometry (Malvern Panalytical Ltd., Malvern, UK). Cu K_α_ radiation (k = 154.05 pm) from a sealed tube was used in the 2Θ angle range 5–90°. For phase analysis, the PANalytical HighScore Plus software package (ver. 4.9) was used combined with the International Centre for Diffraction Data’s powder diffraction file (ICDD PDF-2 ver. 2020) database of standard reference materials. The TPD-CO_2_ measurements were performed in a quartz microreactor using CO_2_ as a probe molecule. In the next step, the CO_2_ was adsorbed on the catalyst surface at 50 °C for 30 min after drying in flowing helium at 400 °C for 60 min. TPD-CO_2_ measurements were carried out in the temperature range 100–600 °C, after removing physisorbed CO_2_ from the catalyst surface. FTIR measurements were done using a Nicolet iS50 FT-IR spectrometer equipped with a liquid-nitrogen cooled MCT detector. A resolution of 4.0 cm^−1^ was used throughout the investigation. A total of 64 scans were taken to achieve a satisfactory signal-to-noise ratio. The background spectrum was collected at 50 °C in an argon stream. Before the measurements, argon was shifted to a mixture of 5 vol % CH_3_OH in the argon stream. The adsorption process involved exposure of the heated catalysts to 5 vol % CH_3_OH in an argon stream flowing at 40 cm^3^/min for 15 min under atmospheric pressure. After the adsorption process, the cell was evacuated for 15 min in an argon stream, and then the spectrum was collected. FTIR spectra of adsorbed species were taken after exposure of the catalysts (10% MgO/NZ, 10% CaO/NZ and 10% SrO/NZ) calcined in an air atmosphere at 400 °C for 4 h or natural zeolite reduced in a mixture of 5% H_2_–95% Ar at 500 °C, to a mixture of 5 vol % methanol–95% argon mixture at 50 °C and after the evacuation of this mixture for 15 min using argon stream.

### 2.3. Catalytic Activity Measurements in Transesterification of the Vegetable Oil with Methanol

The transesterification process was carried out in an autoclave using a substrate mixture of rapeseed oil and CH_3_OH with a molar ratio of 9/1. In all catalytic tests, the weight of about 0.5 g was used. The reaction was performed at 260 °C for 2 h. Before the reaction tests, the catalysts were calcined at 400 °C for 4 h and reduced to 300 °C in a mixture of 5% H_2_–95% Ar. The obtained reaction products were analyzed by the HPLC technique (Shimadzu). In all catalytic tests, column C-18 and as eluent were used as a mixture of 2-isopropanol-hexane (4/5) and methanol. The mobile phase gradient applied during each experiment is shown in Table 1. The reaction products were analyzed using a DAD detector (wavelength: λ = 205 nm) to define triglycerides conversion and for identification of FAME yield.

## 3. Results and Discussion

### 3.1. Transesterification of Vegetable Oil with Methanol Reaction

In this work, biodiesel production via transesterification of vegetable oil with methanol was studied using natural zeolites activated under various conditions. In addition, various metal-oxide catalysts calcined at 400 °C for 4 h were also investigated in the transesterification reaction. Activation of the natural zeolite was performed to improve the biodiesel production process’s catalytic properties. The reactivity results of natural zeolite and metal-oxide catalysts expressed as triglycerides (TG) conversion values, and fatty acids methyl esters yield are given in Table 2. All reaction tests were done using an autoclave with continuous stirring of the reaction mixture. Each process was carried out for 2 h to obtain the final product composed of a mixture of fatty acid alkyl esters, known as biodiesel. The activity measurements obtained for natural zeolite showed that increasing the reaction temperature from 200 to 300 °C results in increased triglyceride conversion and the efficiency of formation of methyl esters of higher fatty acids (FAME yield). To study the effect of the natural zeolite treatment on the catalytic activity, two reaction temperatures of 200 and 220 °C were selected.

The natural zeolite was reduced for 1 h in a mixture of 5% H_2_–95% Ar at various temperatures (300, 400 and 500 °C) or calcined in an air atmosphere at 400 °C for 4 h before catalytic activity tests.

The catalytic activity tests performed for natural zeolite clearly show that calcination or activation at 300 or 400 °C did not improve the catalytic activity of the investigated material. Activation of the natural zeolite at 500 °C resulted in a significant increase in triglyceride conversion value for the process carried out at 200 °C. A natural zeolite activated in a reduction mixture (5% H_2_–95% Ar) performed at 500 °C exhibited a TG conversion value of 54.6% and a FAME yield of 35.6%. In contrast, the same system without activation exhibited a TG conversion value of 37.8% and a FAME yield of 13.1%. It is also worth mentioning that the same system activated in a reduction mixture of 5% H_2_–95% Ar at 500 °C tested in the transesterification process at 220 °C of vegetable oil with methanol exhibited practically the same TG convection (63%), but a higher yield of fatty acid methyl ester formation (51.6%).

In the next step, catalytic activity measurements in biodiesel production were done for metal-oxide catalysts with 10 wt% of CaO, MgO and SrO. In all cases, the catalytic activities in the transesterification process were performed for the calcined metal-oxide catalysts. The results are given in Table 2. All metal-oxide catalysts tested in the studied reaction at 220 °C delivered higher TG conversion and FAME yield than the support itself. The metal-oxide catalysts exhibited TG conversion above 92% and FAME yield values above 93%. The FAME yields for these metal-oxide catalysts can be described by the following dependence series 10% CaO/NZ (98.5%) > 10% MgO/NZ (94.0%) > 10% SrO/NZ (93.2%). The conversion values relationship of metal-oxide catalysts were practically the same, i.e., 10% CaO/NZ (92.4%) = 10% SrO/NZ (92.4%) > 10% MgO/NZ (92.1%). The highest value of FAME yield showed 10% CaO/NZ (98.5%) metal-oxide catalyst, which also exhibited the highest total alkalinity compared to the rest of the metal-oxide catalysts. It is also worth mentioning that the values of the FAME yields may be described by the alkalinity of the studied catalysts in the transesterification process. The catalytic activity measurements showed that the TG and FAME yield of the natural zeolite strongly depends on the catalytic material activation process and is directly influenced by the phase composition of the studied system. The activation process applied before catalytic activity tests has a huge impact on the catalytic material composition and its activity during the transesterification process. In addition, for comparison, the analogical activity measurements were performed for the selected 10% MgO catalyst supported on ZSM 5 zeolite characterized by Si/Al ratio equal 23 and 50, respectively. The Activity measurements showed that for the 10% MgO/ZSM–5 (Si/Al = 23) system, we observed lower TG conversion (91.9) and FAME yield (86.2) compared to the same catalyst 10% MgO supported on the NZ system. It is also worth noticing that in the case of 10% MgO/ZSM–5 (Si/Al = 50) catalyst, we obtained higher TG conversion equal to 94.3%, but a lower FAME yield of 88.9%.

### 3.2. The Characterization of the Physicochemical Properties of the Investigated Catalysts

#### 3.2.1. Basic Properties of the Synthesized Catalyst Systems

CO_2_-TPD experiments were used to characterize the alkalinity of the catalytic material to explain the observed catalytic activity in the biodiesel production process. All catalytic materials were previously heated at 600 °C before a proper alkalinity test. The obtained results for all catalytic systems are given in Table 3. The presented results show that all the investigated metal-oxide catalysts systems exhibited three desorption centers. The calculated values for all investigated materials confirmed that the highest total basicity value among all systems showed 10% CaO/NZ calcined catalyst. This system also exhibited the highest TG conversion and FAME yield values than the rest of the tested catalytic systems.

The pristine natural zeolite itself exhibited the lowest total alkalinity and showed the lowest TG conversion and FAME yield values compared to the rest of the catalytic materials tested in the biodiesel production process. These results are consistent with the activity results obtained for the investigated catalytic systems and agree well with published studies [7,8,38,39,40].

#### 3.2.2. Phase Composition Studies of Metal-Oxide Catalysts

**Figure 1:**
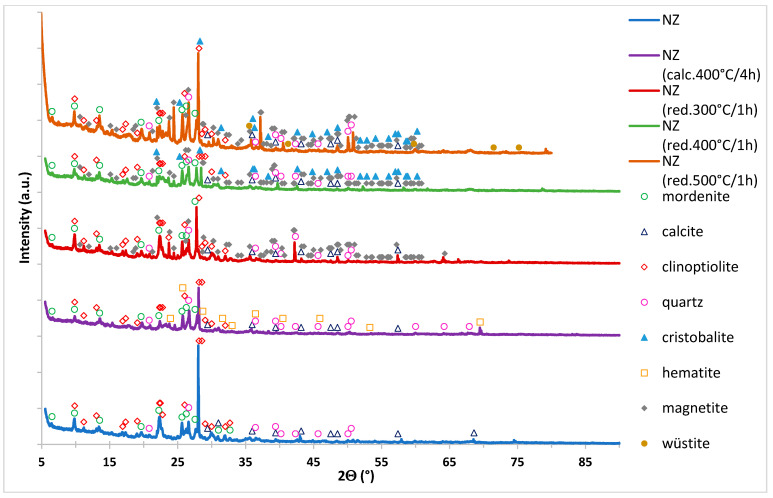
XRD analysis of the pristine natural zeolite and NZ zeolite after various treatment conditions.

**Figure 2:**
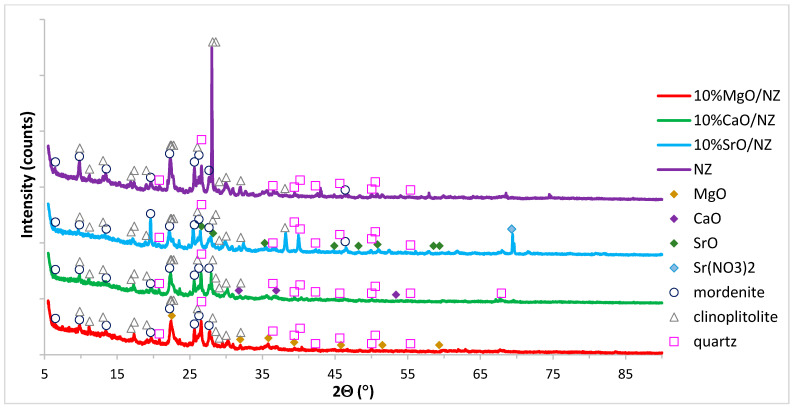
XRD analysis of the calcined in an air atmosphere at 400 °C for 4 h of metallic oxide catalysts supported on natural zeolite.

Conventional XRD measurements were carried out for the support material and the metal-oxide catalysts; the results are given in Figure 1 and Figure 2. Figure 1 presents the influence of the carrier material treatment on the phase composition. As we can easily see from the diffraction curves recorded for the investigated materials, the natural zeolite structure contains calcite, mordenite, clinoptilolite (zeolite) and quartz [9]. The obtained diffraction curve for NZ with the identified crystal structures is presented in Figure 1. The natural zeolite calcination process resulted in the appearance of an additional crystallographic phase of hematite on the diffraction curve (α-Fe_2_O_3_).

The XRD analysis of the NZ system activated in a mixture of 5% H_2_–95% Ar mixture at 300 °C showed the presence of calcite, mordenite, clinoptilolite (zeolite), quartz and magnetite phases. Increasing the reduction temperature to 400 °C resulted in the appearance of an additional phase of cristobalite. The rest of the reflexes observed on the diffraction curve recorded for these systems are assigned to the same crystallographic phases, which were visible in the XRD pattern of the system activated at 300 °C. The analysis of the same systems but activated at higher temperatures (500 °C) confirmed the reduction process of the iron oxide (magnetite) to metallic iron. The rest of the diffraction peaks were assigned to the same phases, which were visible in the case of the system reduced to 400 °C. The phase composition studies of metal-oxide catalysts CaO, MgO and SrO, are given in the same Figure 2. We can easily see from the presented diffraction curves that all calcined metal-oxide catalysts contain mordenite, clinoptilolite and clinoptilolite (zeolite) crystallite structures. In addition, the metal oxide phases of MgO, CaO and SrO were visible on the XRD patterns for the corresponding catalyst composition. It is also worth noting that in the case of the 10% SrO/NZ catalyst, a reflex attributed to the strontium nitrate phase was also observed. This means that in this case, the whole quantity of strontium nitrate was not decomposed during the calcination step.

To elucidate the differences in the catalytic activity of metal-oxide catalysts in biodiesel production reaction, the size of the metal oxide phase of the active phase component of the studied catalysts was investigated. The mean crystallite size was calculated based on Scherrer equation [41]:

D_hkl_ = kλ/βcosθ

where D is the mean dimension of the crystallite perpendicular to the plane (hkl); β is the integral, full widths at half maximum (FWHM) in radians, K = 0.89 is a constant dependent on the crystallite shape, and λ is the X-ray wavelength. The diffraction patterns were processed using HighScore Plus software by fitting to pseudo-Voigt function. Determining the size of the crystallites was quite difficult due to the large number of peaks originating from the natural zeolite. To avoid peak overlap problems, the diffraction patterns were carefully searched, and the following peaks were selected for analysis: MgO (211), CaO and SrO (200) at 2θ angles ca. 39.1°, 37.3° and 35.6°, respectively. The results of the calculation performed for the calcined catalysts are given in Table 4. The analysis of the selected diffraction peaks showed that the lowest crystallite size of metal oxide was observed for the calcined 10% CaO/Nz catalyst. It is also worth mentioning that this catalytic system also exhibited the highest activity and yield of FAME production compared to the rest of the investigated systems. The rest of the catalytic systems containing 10% MgO or 10% SrO oxides showed very similar sizes of the metal oxide crystallites. These catalytic systems also exhibited similar TG conversion and yield of FAME. These results clearly show that the crystallites size of the active phase component is one of the main factors influencing the catalyst activity and selectivity in the transesterification reaction.

#### 3.2.3. Morphology of the Investigated Catalysts

**Figure 3:**
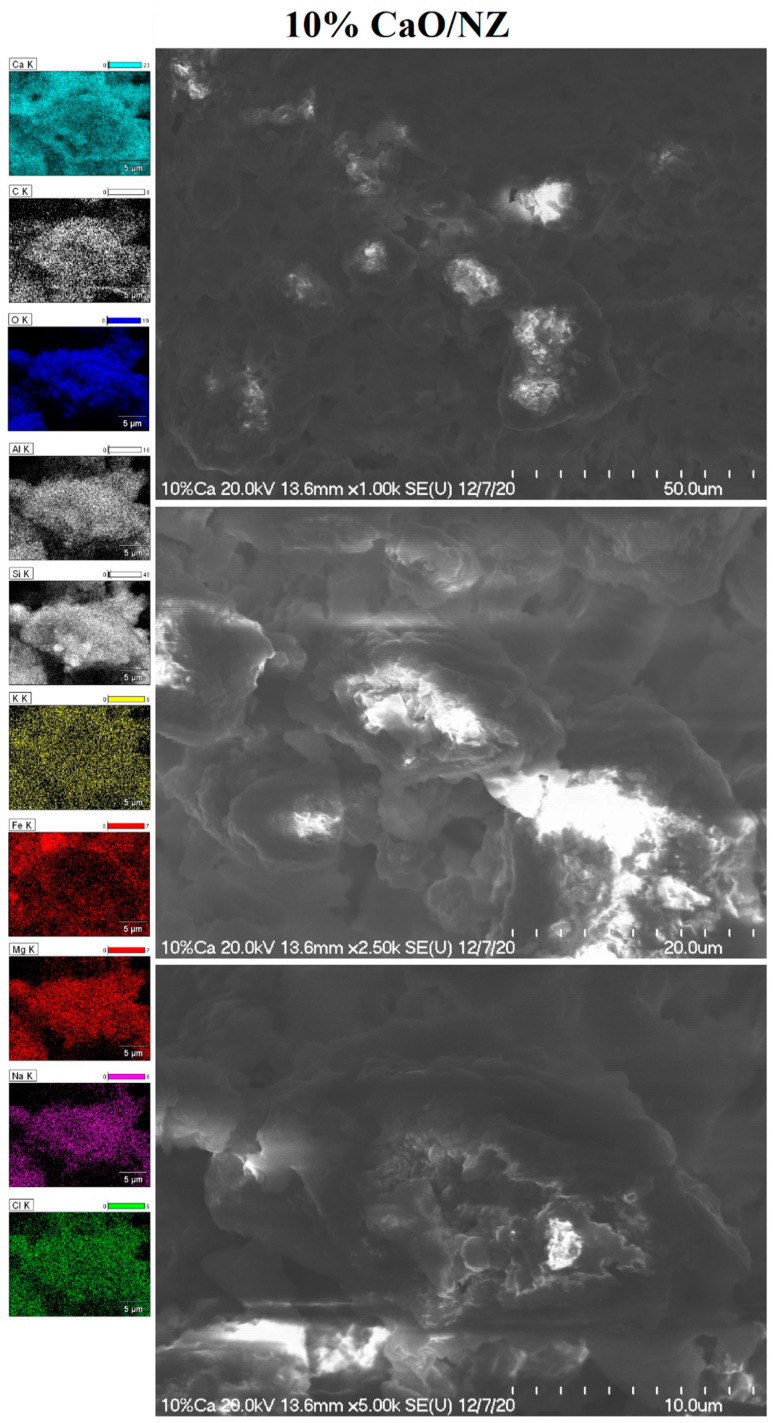
SEM-EDS images of the calcined metal oxide 10% CaO/NZ catalyst in an air atmosphere at 400 °C for 4 h.

**Figure 4:**
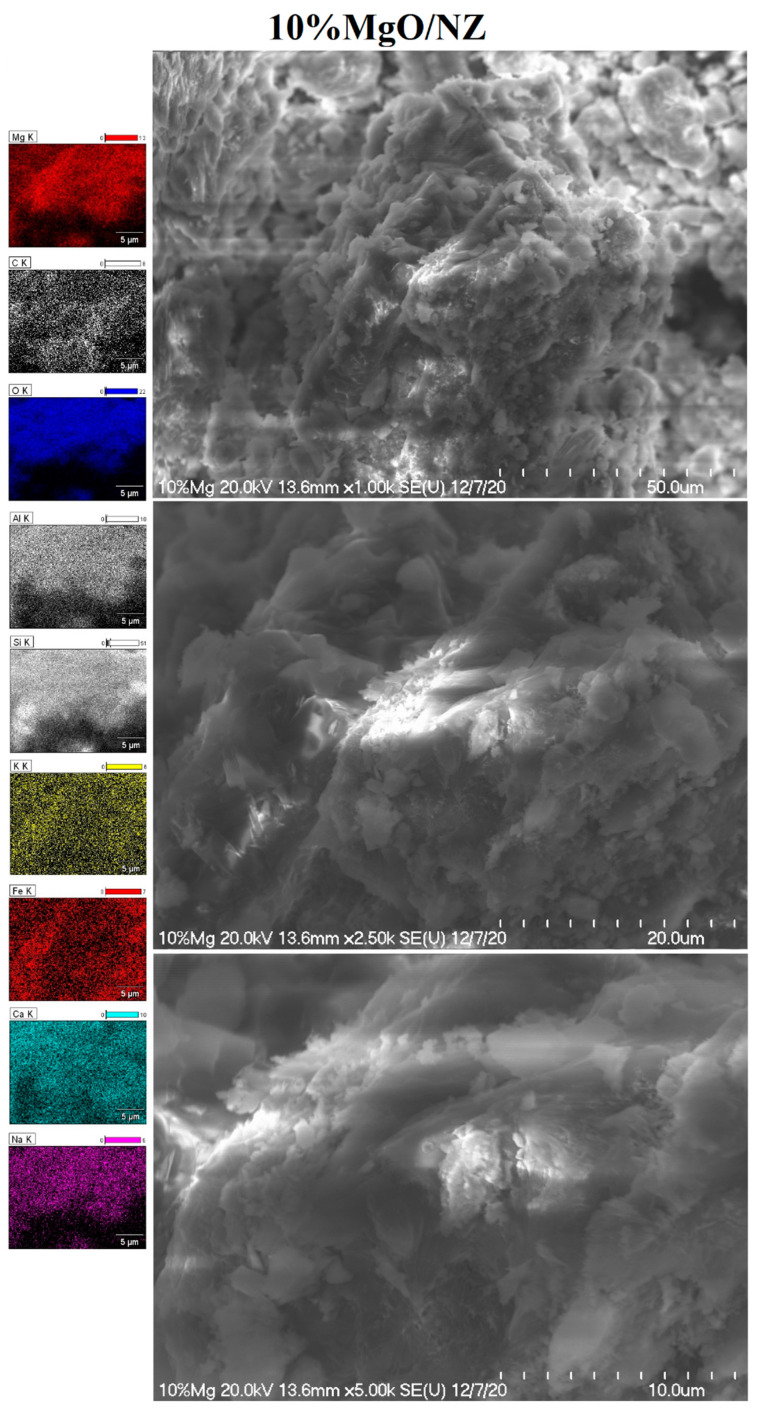
SEM-EDS images of the calcined metal oxide 10% MgO/NZ catalyst in an air atmosphere at 400 °C for 4 h.

**Figure 5:**
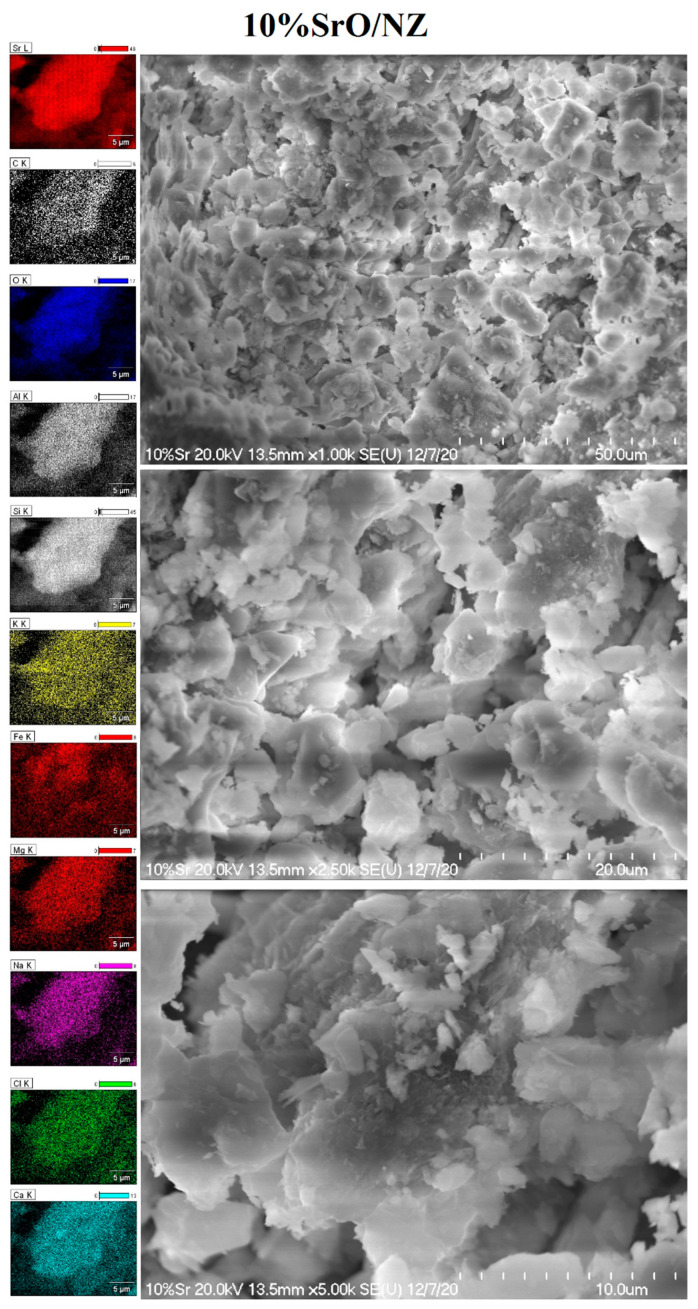
SEM-EDS images of the calcined metal oxide 10% SrO/NZ catalyst in an air atmosphere at 400 °C for 4 h.

SEM-EDS measurements were done for the metallic oxide catalysts supported on natural zeolite. Scanning electron microscopy with an EDS detector is a suitable method, which allows determining the morphology and the catalyst surface composition. SEM images for all investigated catalysts were collected from the surface of the catalyst, and the obtained results are given in Figure 3, Figure 4 and Figure 5, respectively. The surface analysis of the 10% CaO/NZ catalyst confirmed the presence of Ca, C, O, Al, Si, K, Fe, Mg, Na and Cl on the surface. The presence of these elements confirmed the presence of the support components and active phase elements. The same surface analysis was performed for the 10% MgO/NZ system, and the results are given in Figure 4. The results from the SEM-EDS measurements showed that the composition of the magnesium oxide catalysts was the same as in the calcium oxide supported catalyst. The only difference in the obtained SEM-EDS results obtained for the MgO/NZ catalyst was the lack of surface chlorine, the presence of which was confirmed on the surface of the 10%CaO/NZ catalyst. Analogical SEM-EDS measurements were also performed for SrO metallic oxide supported catalysts, and the results are given in the next Figure 5. The analysis of this catalyst surface showed that all catalysts contain the same elements detected for the previously investigated catalysts. Identified elements on the 10% SrO/NZ catalyst surface confirmed the composition of the investigated catalyst and the presence of Cl, which comes from the precursor salt used during the preparation step. In addition, it is also worth noting that the presence of all identified elements agrees well with the XRD results in the case of the calcined supported catalyst. In addition, SEM-EDS results gave evidence that the CaO catalyst was characterized by the most homogeneous distribution of calcium on the surface. In comparison, in the case of Mg- and Sr-containing systems, one can see the regions with higher Mg and Sr content on the support surface. This behavior is typical for catalysts prepared using a wet impregnation method and may explain the differences in the catalytic activity of the tested systems in the transesterification reaction. In addition, the observed catalytic activity results showed that in the case of the most active calcium oxide catalyst, the lowest intensity of the diffraction peaks assigned to the calcium oxide phase were detected by the conventional XRD method, which agrees well with the results calculated from Scherrer’s equations. These results may explain the differences in the catalytic activity of the tested catalytic material in the biodiesel production process.

#### 3.2.4. Sorption Properties of Metal-Oxide Catalysts and Natural Zeolite in Relation to Methanol

**Figure 6:**
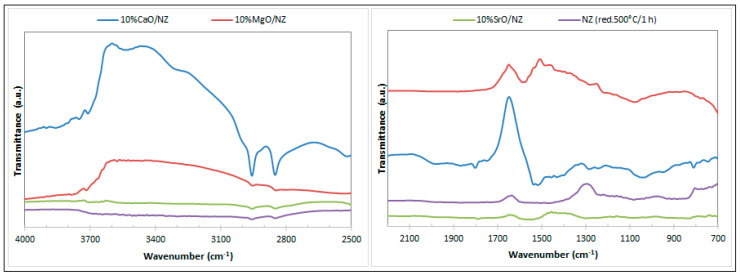
FTIR spectra of adsorbed species taken after exposure of the catalytic system (10% MgO/NZ, 10% CaO/NZ and 10% SrO/NZ) calcined in an air atmosphere at 400 °C for 4 h or natural zeolite reduced in a mixture of 5% H_2_–95% Ar at 500 °C, to a mixture of 5 vol % methanol–95% argon mixture at 50 °C and after the evacuation of this mixture for 15 min using argon stream.

In the next step of the physicochemical studies carried out over metal-oxide catalysts, the sorption properties of MO/NZ (where MO = MgO, CaO and SrO) systems in relation to methanol–argon mixture were investigated. The purpose was to explain and understand the activity of the investigated systems in the biodiesel production process. The results of the FTIR studies are shown in Figure 6. Several characteristic IR bands can be easily distinguished in the spectra recorded for the investigated systems. The IR spectra of all catalyst systems calcined at 400 °C showed visible bands located at 2957, 2823, 1537, 1448 and 1370 cm^−1^. In addition, in all cases, a broad band between 1400 and 1600 cm^−1^ of strongly overlapping OH and CH vibrations was visible on the IR spectra. In the case of the MgO/NZ and CaO/NZ catalysts systems, weak bands around 951–1100 cm^−1^ were visible and were assigned to the CO stretching mode [42]. The broad OH stretching vibration modes in the region of 3237–3384 cm^−1^ and 3482–3586 cm^−1^ were visible in the 10% CaO/NZ catalyst spectrum, and these bands were visible only for the calcium oxide catalyst supported on NZ. The specific bands located at 2957 and 2850 cm^−1^ were assigned to the antisymmetric and symmetric CH stretching vibration bands for all catalyst systems. It should be emphasized that the highest intensity of those bands was observed for the CaO/NZ system, which confirmed the strongest adsorption of methanol on the catalyst surface compared to the rest of the investigated catalysts and support. It is also worth noting that in the case of the IR spectra recorded for the CaO/NZ catalyst, an additional band located at 2910 cm^−1^ was detected, and this band is associated with overtones or combinations [43,44]. In addition, the wide band located on the FTIR spectra above 3666 cm^−1^ for CaO and MgO catalysts was attributed to the symmetric OH stretching vibration modes. FTIR measurements carried out for all investigated systems allow concluding that the greatest quantity of adsorbed methanol was observed for 10% CaO/NZ catalyst calcined in an air atmosphere for 4 h at 400 °C. The FTIR spectra of all studied systems gave evidence that the quantity of methoxide species adsorbed on the catalyst surface is one of the first steps of the transesterification process over the catalyst surface and plays an important role in the studied process [17,45]. This observation explains the activity row in the studied reaction and agrees with our insights from the results obtained using other research techniques.

## 4. Conclusions

This work presents the catalytic and physicochemical properties of metal-oxide catalysts supported on natural zeolite in the transesterification reaction. The influence of the activation process of the NZ systems on their reactivity properties in biodiesel production was also investigated in this work. The obtained results showed that the phase composition of the NZ itself has a strong influence on the catalytic activity of the NZ system in biodiesel production. It was proven that the pretreatment of the NZ catalysts, especially the reduction process, leads to the improvement of the catalytic activity, which is explained by the change in the phase composition of the tested materials. The catalytic results clearly showed that the metallic iron particles are active in TG conversion via the transesterification process. The activity results clearly showed that NZ systems reduced in a reduction mixture at 500 °C exhibited higher TG conversion value and FAME yield in the biodiesel production process performed at both 200 and 220 °C. The reactivity results showed that the promotion of the NZ system by metal oxides improves the catalytic activity, which can be explained by the generation of additional active centers by introducing metal oxides onto the surface of the natural zeolite. The highest activity expressed as TG conversion and selectivity to FAME exhibited the 10% CaO/NZ catalyst compared to the rest of the tested catalytic systems. The highest TG conversion and FAME yield values are explained by the highest alkalinity, the largest dispersion of CaO on the support surface, and the strongest sorption of methanol on its surface compared to the other systems studied.

## Figures and Tables

**Table 1:** Phase gradient used in the HPLC measurements.

Mobile Phase Gradient			Flow Rate mL·min^−1^
Time (min)	Solvent A (%)	Solvent B (%)	
0.0	100	0	1
20.0	100	0	1
45.0	0	100	1
70.0	0	100	1
75	100	0	1

Solvent A: methanol; solvent B: 2-propanol/hexane = 4/5; injection volume: 1 μL; column temperature: 25 °C.

**Table 2:** The results of the transesterification process performed over pristine, treated NZ (reduced in a mixture of 5% H_2_–95% Ar, calcined in an air atmosphere at 400 °C for 4 h) and metal-oxide catalysts.

Catalyst	Reaction Temperature (°C)	Reaction Time (h)	Molar Ratio Oil: Methanol	Catalyst Weight (g)	Triglycerides Conversion (%)	FAME Yield (%)
NZ	200	2	1:9	0.5	37.8	13.1
NZ	220	2	1:9	0.5	63.3	44.2
NZ	240	2	1:9	0.5	76.5	65.8
NZ	260	2	1:9	0.5	90.5	67.2
NZ	280	2	1:9	0.5	93.2	84.7
NZ	300	2	1:9	0.5	94.5	87.3
NZ (calc. 400 °C/4 h)	200	2	1:9	0.5	29.6	22.3
NZ (calc. 400 °C/4 h)	220	2	1:9	0.5	54.0	46.6
NZ (red. 300 °C/1 h)	200	2	1:9	0.5	36.0	17.5
NZ (red. 300 °C/1 h)	220	2	1:9	0.5	59.9	42.9
NZ (red. 400 °C/1 h)	200	2	1:9	0.5	36.2	28.0
NZ (red. 400 °C/1 h)	220	2	1:9	0.5	60.3	44.0
NZ (red. 500 °C/1 h)	200	2	1:9	0.5	54.6	35.6
NZ (red. 500 °C/1 h)	220	2	1:9	0.5	63.0	51.6
10% CaO/NZ (calc. 400 °C/4 h)	220	2	1:9	0.5	92.4	98.5
10% MgO/NZ (calc. 400 °C/4 h)	220	2	1:9	0.5	92.1	94.0
10% MgO/ZSM–5 (Si/Al = 23)	220	2	1:9	0.5	91.9	86.2
10% MgO/ZSM–5 (Si/Al = 50)	220	2	1:9	0.5	94.3	88.9
10% SrO/NZ (calc. 400 °C/4 h)	220	2	1:9	0.5	92.4	93.2

**Table 3:** The amount of CO_2_ adsorbed on the support surface (calcined in an air atmosphere for 4 h at 400 °C), metal oxide calcined catalysts, calculated from the CO_2_-TPD profiles.

Catalyst	Total Basicity (mmol/g)	Weak Centers (100–300 °C) (mmol/g)	Medium Centers (300–450 °C) (mmol/g)	Strong Centers (450–600 °C) (mmol/g)
NZ	0.84	0.42	0.03	0.39
10% CaO/NZ	2.41	0.11	0.12	2.17
10% MgO/NZ	1.48	0.08	0.09	1.32
10% SrO/NZ	1.29	0.29	0.18	0.82

**Table 4:** Size of metal oxide crystallites estimated by the Scherrer method for the selected peaks from XRD measurements performed for metal-oxide catalysts calcined in an air atmosphere at 400 °C for 4 h.

No.	Catalysts	The Size of MO Crystallites [nm]
**1.**	10% CaO/NZ	CaO = 35(2)
**2.**	10% MgO/NZ	MgO = 83(1)
**3.**	10% SrO/NZ	SrO = 91(2)

## Data Availability

The data presented in this study are available on request from the corresponding author.
